# MiR-30a-5p Overexpression May Overcome EGFR-Inhibitor Resistance through Regulating PI3K/AKT Signaling Pathway in Non-small Cell Lung Cancer Cell Lines

**DOI:** 10.3389/fgene.2016.00197

**Published:** 2016-11-15

**Authors:** Fei Meng, Fengfeng Wang, Lili Wang, S. C. Cesar Wong, William C. S. Cho, Lawrence W. C. Chan

**Affiliations:** ^1^Department of Health Technology and Informatics, The Hong Kong Polytechnic UniversityHong Kong, China; ^2^Department of Clinical Oncology, Queen Elizabeth HospitalHong Kong, China

**Keywords:** EGFR, IGF-1R, microRNA, PI3K/AKT signaling pathway, drug resistance, non-small cell lung cancer

## Abstract

Lung cancer is one of the most common deadly diseases worldwide, most of which is non-small cell lung cancer (NSCLC). The epidermal growth factor receptor (EGFR) mutant NSCLCs frequently respond to the EGFR tyrosine kinase inhibitors (EGFR-TKIs) treatment, such as Gefitinib and Erlotinib, but the development of acquired resistance limits the utility. Multiple resistance mechanisms have been explored, e.g., the activation of alternative tyrosine kinase receptors (TKRs) sharing similar downstream pathways to EGFR. MicroRNAs (miRNAs) are short, endogenous and non-coding RNA molecules, regulating the target gene expression. In this study, we explored the potential of miR-30a-5p in targeting the EGFR and insulin-like growth factor receptor-1 (IGF-1R) signaling pathways to overcome the drug resistance. IGF-1R is one of the tyrosine kinase receptors that share the same EGFR downstream molecules, including phosphatidylinositol 3 kinase (PI3K) and protein kinase B (AKT). In this work, an *in vitro* study was designed using EGFR inhibitor (Gefitinib), IGF-1R inhibitor (NVP-AEW541), and miRNA mimics in two Gefitinib-resistant NSCLC cell lines, H460 and H1975. We found that the combination of EGFR and IGF-1R inhibitors significantly decreased the phosphorylated AKT (p-AKT) expression levels compared to the control group in these two cell lines. Knockdown of phosphoinositide-3-kinase regulatory subunit 2 (PIK3R2) had the same effect with the dual inhibition of EGFR and IGF-1R to reduce the expression of p-AKT in the signaling pathway. Overexpression of miR-30a-5p significantly reduced the expression of the PI3K regulatory subunit (PIK3R2) to further induce cell apoptosis, and inhibit cell invasion and migration properties. Hence, miR-30a-5p may play vital roles in overcoming the acquired resistance to EGFR-TKIs, and provide useful information for establishing novel cancer treatment.

## Introduction

Lung cancer, particularly non-small cell lung cancer (NSCLC), is one of the top cancers leading to death worldwide. Every year there are more than 4000 new cases and the 5-year survival rate is around 15% only (Ho, 2011). The early detection is applied to clinical screening on a large scale and most of patients are diagnosed with NSCLCs (Breuer et al., 2005). However, the survival rate is still very low. In order to improve the survival, much attention has been paid to explore the new therapeutic approaches focusing on the molecular mechanisms that regulate tumor cell growth, such as the target therapy on epidermal growth factor receptor (EGFR; Camp et al., 2005). EGFR tyrosine kinase inhibitors (TKIs) are small molecules that bind to the tyrosine kinase domain of EGFR to inhibit its phosphorylation and the subsequent receptor activation and signal transduction (Camp et al., 2005). However, tumors usually progress due to the acquired resistance to EGFR-TKIs (Yu et al., 2013). No curative therapy is available for NSCLC patients with such resistance, even though the irreversible EGFR-TKIs and other novel agents are under development (Zhang et al., 2012; Yu et al., 2013). Multiple molecular mechanisms of resistance have been explored, such as the activation of alternative tyrosine kinase receptors (TKRs) that bypass the EGFR signaling pathway, and the secondary mutations in EGFR T790M (Camp et al., 2005; Zhang et al., 2012).

Researchers found that other TKRs are activated, including hepatocyte growth factor receptor (c-MET) and insulin-like growth factor receptor-1 (IGF-1R), which share similar downstream pathways with EGFR (Camp et al., 2005). The activation of these alternative TKRs could mimic the function of EGFR by regulating the overlapping signal transduction pathways to stimulate the survival mechanisms (Camp et al., 2005). IGF-1R is an important tyrosine kinase that is involved in malignant transformation and acquired resistance of many human cancers (Guevara-Aguirre et al., 2011; Chan et al., 2015). It has been reported that the activation of IGF-1R is related to EGFR-TKIs resistance in NSCLC cell lines and lung cancer patients (Peled et al., 2013; Yeo et al., 2015). Researchers also found that IGF-1R tyrosine kinase inhibitors can reverse the resistance of EGFR-TKIs *in vitro* and *in vivo* (Morgillo et al., 2006; Zhou et al., 2015). Evidences suggest a role of IGF-1R in the progression of tumor and a functional cross talk between EGFR and IGF-1R (Tandon et al., 2011). Double inhibiting EGFR and IGF-1R may be a potential strategy to reverse the drug resistance against the 2nd or 3rd generation EGFR inhibitors (Chan et al., 2015). However, the exact mechanisms of IGF-1R-induced acquired drug resistance in NSCLC remain unclear.

MicroRNAs (miRNAs) are short, endogenous and non-coding RNA fragments of 21–25 nucleotides, which can inhibit mRNA translation or promote mRNA degradation (Yeo et al., 2015). MiRNAs are very important in human tumors for altering the expression of target oncogenes or tumor suppressor genes (Zhou et al., 2015). The dysregulation of miRNA contributes much to cancer development and cancer drug resistance (Morgillo et al., 2006; Yeo et al., 2015). Researchers reported that miR-30b, miR-30c, miR-221, and miR-222 affect the Gefitinib-induced apoptosis and epithelial-mesenchymal transition of NSCLC cells by inhibiting some important oncogenes expression, e.g., sarcoma viral oncogene homolog (SRC; Yeo et al., 2015). Furthermore, miR-494 can alter the drug resistance by regulating the expression of Bcl-2-like protein 11 (BIM; Tandon et al., 2011). The regulatory effects of miRNAs on EGFR signaling pathway in NSCLCs have been widely studied. MiR-128b directly regulates the expression of EGFR, whose loss-of-heterozygosity is frequently found in NSCLCs and promotes the clinical response and survival rate after Gefitinib treatment (Davalos et al., 2012). Moreover, miR-7 regulates various genes in the EGFR signaling pathway, including EGFR, Raf1, AKT, and ERK, indicating that miR-7 could inhibit the EGFR signaling pathway (Pao et al., 2005). As such, miRNAs are important to affect the resistance to EGFR-TRIs in the EGFR signaling pathway.

IGF-1R can activate the EGFR shared downstream signaling pathways, one of which is the phosphatidylinositol 3 kinase/protein kinase B (PI3K/AKT) pathway (Zhou et al., 2015). In this study, we selected two key markers in the shared downstream signaling pathways PI3K and AKT to verify the effect of a candidate miRNA miR-30a-5p on the drug resistance in NSCLCs. MiR-30a-5p is predicted to regulate phosphoinositide-3-kinase regulatory subunit 2 (PIK3R2) by TargetScan (Agarwal et al., 2015) and PicTar (Krek et al., 2005) prediction databases. Furthermore, our previous study revealed that miR-30a-5p was negatively associated with the expression of PIK3R2 based on the multiple linear regression and support vector regression models (Wang et al., 2016). We hypothesize that inhibition of the shared downstream molecules (e.g., PI3K) of EGFR and IGF-1R can overcome the resistance of EGFR-TKIs. MiRNAs regulating the shared downstream molecules have the same effect to reverse the drug resistance. To prove our hypothesis, an *in vitro* study was performed using EGFR inhibitor, IGF-1R inhibitor, and miRNA mimics in two Gefitinib-resistant NSCLC cell lines, NCI-H1975 with a secondary T790M mutation in EGFR, and NCI-H460. This study could identify miRNA roles in overcoming the acquired resistance to EGFR-TKIs, and explore new mechanisms of NSCLCs.

## Materials and methods

### Cell culture and reagents

The cell lines NCI-H460 and NCI-H1975 were purchased from the Type Culture Collection of the Chinese Academy of Sciences (Shanghai, China). Cells were grown in RPMI-1640 supplemented with 10% fetal bovine serum (FBS), and incubated at 37°C, 5% CO_2_. The EGFR inhibitor Gefitinib (#4765) was purchased from Cell Signaling Technology. The IGF-1R inhibitor NVP-AEW541 (#S1034) was purchased from Selleckchem. RIPA lysis and extraction buffer (#89900) used to lyse cells was purchased from ThermoFisher. PIK3R2-shRNA (#sc-39125-SH) and the Control shRNA Plasmid-A (#sc-108060) were purchased from Santa Cruz Biotechnology. MiR-30a-5p mimics (5′-UGUAAACAUCCUCGACUGGAAG-3′) and the negative control RNA oligo (5′-UUCUCCGAACGUGUCACGUTT-3′) were purchased from GenePharma. PIK3R2-shRNA and miR-30a-5p mimics were transfected by lipofectamine 2000 reagent (#12566014) purchased from ThermoFisher. Annexin V-FITC Apoptosis Detection Kit (#K101-25) was purchased from BioVision. The CytoSelect™ Cell Invasion Assay Kit (#CBA-110) was purchased from CELL BIOLABS, INC.

### Western-blotting and antibodies

Protein fractions were extracted from cells. Cells were washed twice in Phosphate-buffered saline (PBS). We then scraped the cells from plates using tips. Samples were homogenized in ice by the addition of 400 μl RIPA lysis buffer containing 1 mM sodium ortho vanadate and protease inhibitors cocktail, and then maintained constant agitation for 30 min at 4°C. The lysates were collected by centrifugation at 13000 rpm for 20 min at 4°C. Protein concentration was measured by Bradford assay (Coomassie Protein Assay, Pierce, Rockford, IL, USA), and the bovine serum albumin (BSA) was used as the standard. Thirty micrograms/lane of protein was loaded on 8% polyacrylamide gel and subjected to electrophoretic separation by sodium dodecyl sulfate-polyacrylamide gel electrophoresis (SDS–PAGE). After that, proteins were transferred to polyvinylidene difluoride (PVDF) membranes (Immobilon P, Millipore, Billerica, MA, USA) and probed with specific primary antibodies from Cell Signaling Technology, USA (EGF Receptor (D38B1) XP® Rabbit mAb, #4267, 1:1000; Phospho-EGF Receptor (Tyr1068) (D7A5) XP® Rabbit mAb, #3777, 1:1000; IGF-I Receptor β (D23H3) XP® Rabbit mAb, #9750, 1:1000; Phospho-IGF-I Receptor β (Tyr1135/1136)/Insulin Receptor β (Tyr1150/1151) (19H7) Rabbit mAb, #3024, 1:1000; PI3 Kinase p85 Antibody, #4292, 1:1000; AKT (pan) (C67E7) Rabbit mAb, #4691, 1:1000; Phospho-AKT (Ser473) (D9E) XP® Rabbit mAb, #4060, 1:2000; GAPDH (D16H11) XP® Rabbit mAb, #5174, 1:2000), and then incubated with the appropriate horseradish peroxidase (HRP)-conjugated secondary antibodies (Anti-rabbit IgG, HRP-linked Antibody, #7074, 1:3000). The chemiluminescent was detected by a Kodak 4000R Pro camera. The resulting bands from western blotting were quantified as optical density (OD) × band area and expressed as arbitrary units. GAPDH was probed as the reference of internal control. All the data were normalized to the signal of GAPDH.

### Dual inhibition of EGFR and IGF-1R

Gefitinib and AEW541 were used in combination to test the inhibition effect on the signaling pathway. The concentrations of Gefitinib and AEW541 were formulated according to the manufacturer's instructions. The 10 mM stock of Gefinitib reconstituted the 10 mg power in 2.24 ml DMSO. The 10 mg AEW541 power was dissolved in 2.2751 ml DMSO to form 10 mM stock. The appropriate stocks were took to dilute to 2 μM concentrations in culture medium containing 10% FBS. Cells were seeded in 6-well plates with 70–80% confluency in each well after 24 h incubation, and aspirated the old medium. Cells were then respectively treated with 2 ml prepared medium with Gefitinib, AEW541, and the combination of Gefitinib and AEW541 for 6 h. The expression levels of phosphorylated and total signaling proteins were detected by western blotting.

### Knockdown of PI3K through PIK3R2-shRNA transfection

The PI3K regulatory subunit PIK3R2 was chosen for the knockdown analysis. PIK3R2 knockdown assays were carried out using lipofectamine 2000 kit to transfect PIK3R2-shRNA into cells. Cells were seeded in 6-well plates with 70–80% confluency in each well for 24 h incubation. PIK3R2-shRNA (2.5 μg) in 125 μl of Opti-MEM® I Reduced Serum Medium (#31985062, ThermoFisher) mixed with 5 μl of lipofectamine 2000 transfection reagent dissolved in 125 μl of the same medium and allowed to stand at room temperature for 20 min according to the manufacturer's instructions. After that, the resulting 250 μl transfection solutions were added to each well with 1.75 ml of medium. The cultures were then replaced with 2 ml fresh medium supplemented with 10% FBS after 6 h. The Control shRNA Plasmid-A in the lipofectamine 2000 was added as the negative control group, and only lipofectamine 2000 reagent as the blank control group. After 24 h incubation, cells were lysed to extract proteins and detect the phosphorylated AKT (p-AKT) level by western blotting.

### Overexpression of miR-30a-5p

#### MiR-30a-5p mimics transfection

Cells were seeded in glass-bottom dishes and 6-well plates with 70–80% confluency in each well after 24 h incubation. MiR-30a-5p mimics (75 pmol) in 125 μl of Opti-MEM® I Reduced Serum Medium mixed with 5 μl of lipofectamine 2000 transfection reagent dissolved in 125 μl of the same medium and allowed to stand at room temperature for 20 min according to the manufacturer's instructions. After that, the resulting 250 μl transfection solutions were added to each well with 1.75 ml of medium. The cultures were then replaced with 2 ml fresh medium supplemented with 10% FBS after 6 h. After that, the cells were incubated for 24 h. The RNA oligo in the lipofectamine 2000 was added as the negative control group, and only lipofectamine 2000 reagent as the blank control group. The resulted samples were used for the western blotting, cell apoptosis, invasion, and wound healing assays.

#### Cell apoptosis assay

The cell apoptosis assay was performed using the Annexin V-FITC Apoptosis Detection Kit according to the manufacturer's instructions. The cells in glass-bottom dishes from the resulted sample were washed twice in PBS, and added 500 μl 1 X binding buffer. And then added 5 μl of Annexin V-FITC and 5 μl of propidium iodide, and incubated for 5 min in the dark at room temperature. The microscope was used to detect the signals and take images. Cells bound to Annexin V-FITC show green staining in the plasma membrane, while, cells lost membrane integrity show red staining throughout the nucleus and green staining on the cell surface. The microscope was used to detect the apoptosis signals and take images at 6 and 12 h after adding Annexin V-FITC. We counted the number of apoptotic and total cells under 40X objective in three individual fields per dish for each biological repeat. The cell apoptosis rate was calculated based on the equation: percentage apoptosis rate = apoptotic cell number/total cell number^*^100.

#### Cell invasion assay

We performed the cell invasion assay using CytoSelect™ Cell Invasion Assay Kit according to the manufacturer's instructions. Five-hundred microliters of medium containing 10% FBS was added to the lower well of the invasion plate. Three-hundred microliters of cell suspension solution from the resulted samples with the concentration of 1.0 × 10^6^ cells/ml in serum free medium was then added to the upper chamber of the kit. The cells were incubated for 24 h at 37°C, 5% CO_2_. During this process, the invasive cells went through the basement membrane layer of the kit and attached to the bottom of the insert membrane, while the non-invasive cells stayed in the upper chamber. The non-invasive cells were removed carefully. The invasive cells were transferred to a clean well containing 400 μl of Cell Stain Solution and incubated for 10 min at room temperature. The microscope was used to image the invasive cells. Gently washed the stained cells in 200 μl Extraction Solution and incubated for 10 min. One-hundred microliters of the resulted solution was then transferred to a 96-well microtiter plate and measured the OD 560 nm in a microplate reader.

#### Wound healing assay

The wound healing assay was used to indicate the cell migration ability. A straight scratch was made gently through the monolayer cells using an Eppendorf tip in 6-well plates from the resulted samples. Detached cells were washed away using PBS buffer and the serum-free culture medium was added. Cell migrating to the scratch was monitored and took images at 0, 12, and 24 h after given the wound using microscope. The migration rate was calculated according to the equation: percentage wound healing = ((wound length at 0 h) − (wound length at 12 or 24 h))/(wound length at 0 h)^*^ 100 (Davalos et al., 2012).

### Statistical analysis

The statistical analyses were performed by IBM SPSS Statistics 24.0 software. Three independent experiments were performed for western blotting, cell apoptosis, invasion and wound healing assays. All the experimental values were given as the mean ± standard error of the mean (SEM). One-way ANOVA followed by Tukey's *post-hoc* test was performed to make the comparisons for western blotting. The Student *t*-test was applied to determine the statistical significance in the cell apoptosis, invasion, and wound healing assays. Significant differences were considered at *p* < 0.05.

## Results

### Combination of EGFR and IGF-1R inhibitors blocks the PI3K/AKT signaling pathway

**Figure 1:**
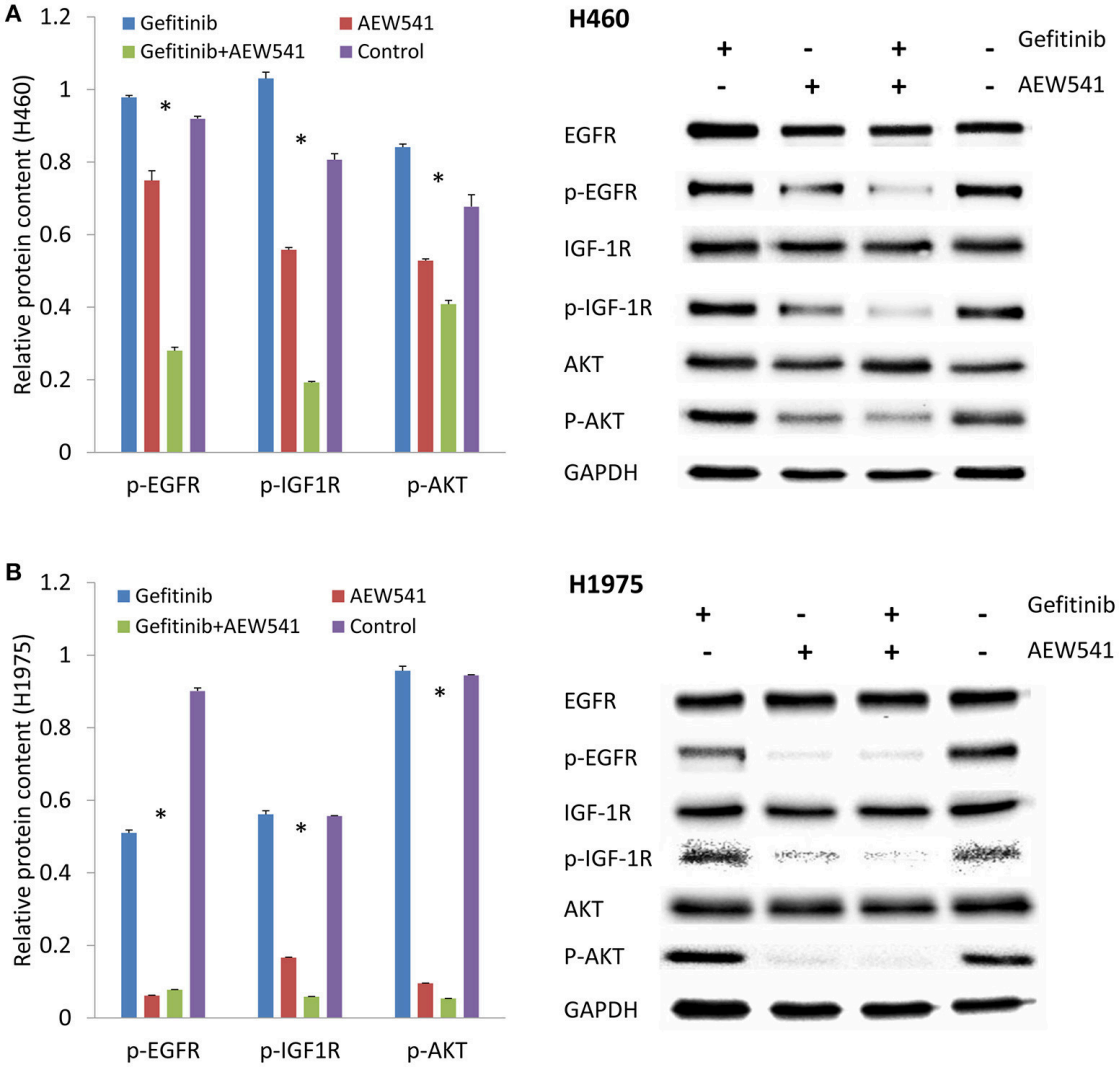
**Effect of EGFR inhibitor (Gefitinib), IGF-1R inhibitor (AEW541), and the combination (Gefitinib+AEW541) on the protein expression levels of total and phosphorylated EGFR, IGF-1R and AKT. (A)** Protein expression in H460 cell line. **(B)** Protein expression in H1975 cell line. GAPDH was used as an internal control. The chart results were presented as the mean of three independent experiments. Blots were the representative of three independent experiments. ^*^P < 0.05, one-way ANOVA used to compare four groups.

H460 and H1975 cells were treated with Gefitinib, AEW541, and the combination of Gefitinib and AEW541, respectively. In order to explore the effects at the mechanistic level, we tested the phosphorylation levels of related proteins after treatment. One-way ANOVA revealed that there were significant differences among the four groups (including three experiment and one control groups) with *p* < 0.05 for the expression levels of p-EGFR, p-IGF-1R, and p-AKT in both H460 and H1975 cells (Figure 1). Interestingly, we identified that IGF-1R inhibitor (AEW541) could affect the expression of p-EGFR in both H460 and H1975 cells. Notably, we found that the combination of Gefitinib and AEW541 inhibitors significantly decreased p-IGF-1R and p-AKT expression levels compared to the only Gefitinib, only AEW541 and control groups from Tukey's *post-hoc* test in both of the two cell lines (*p* < 0.05). AKT is the downstream molecule in the signaling pathways, and the expression level of its phosphorylated form was the least in the dual inhibitors group compared to the other three groups. Hence, the combination of EGFR and IGF-1R inhibitors treatment could block the PI3K/AKT signaling pathway.

### Treatment with PI3K-shRNA decreases the expression level of p-AKT

**Figure 2:**
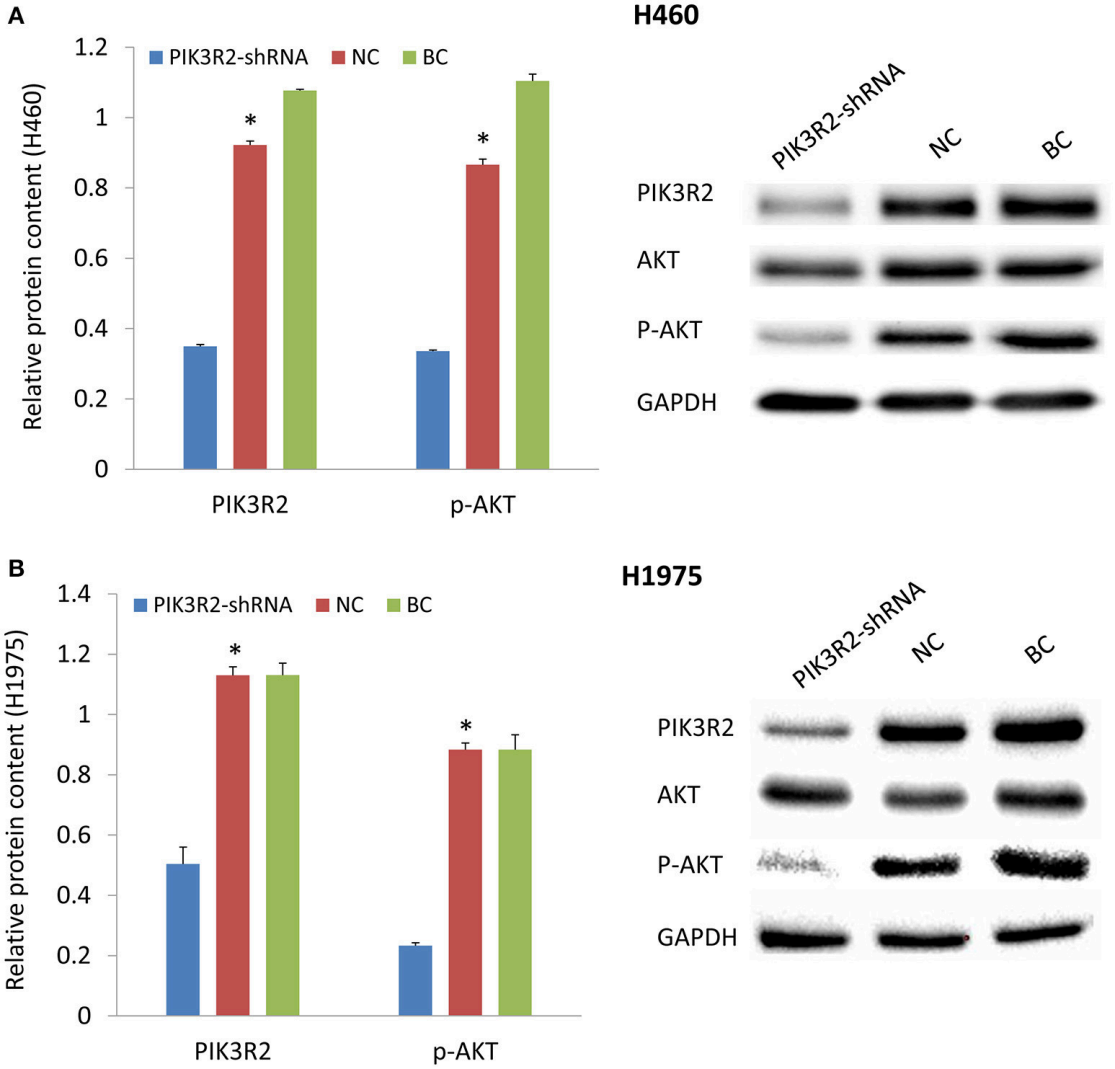
**Effect of PIK3R2-shRNA knockdown on the protein expression levels of PIK3R2 and AKT**. **(A)** Protein expression in H460 cell line. **(B)** Protein expression in H1975 cell line. GAPDH was used as an internal control. The chart results were presented as the mean of three independent experiments. Blots were the representative of three independent experiments. ^*^P < 0.05, one-way ANOVA used to compare three groups.

PIK3R2-shRNA was transfected into H460 and H1975 cells. The results demonstrated that the PIK3R2 expression level was significantly decreased after the transfection compared to the negative and blank control groups from Tukey's *post-hoc* test (*p* < 0.05; Figure 2). The expression level of its phosphorylated downstream molecule p-AKT was also significantly decreased in the shRNA knockdown group compared to the control groups (*p* < 0.05). PI3K is the shared downstream molecule of EGFR and IGF-1R. Inhibition of PI3K regulatory subunit PIK3R2 could decrease the expression level of p-AKT and block the subsequent signaling pathway in both H460 and H1975 cells.

### Treatment with miR-30a-5p mimics decreases the expression levels of PIK3R2 and p-AKT and induces cell apoptosis

**Figure 3:**
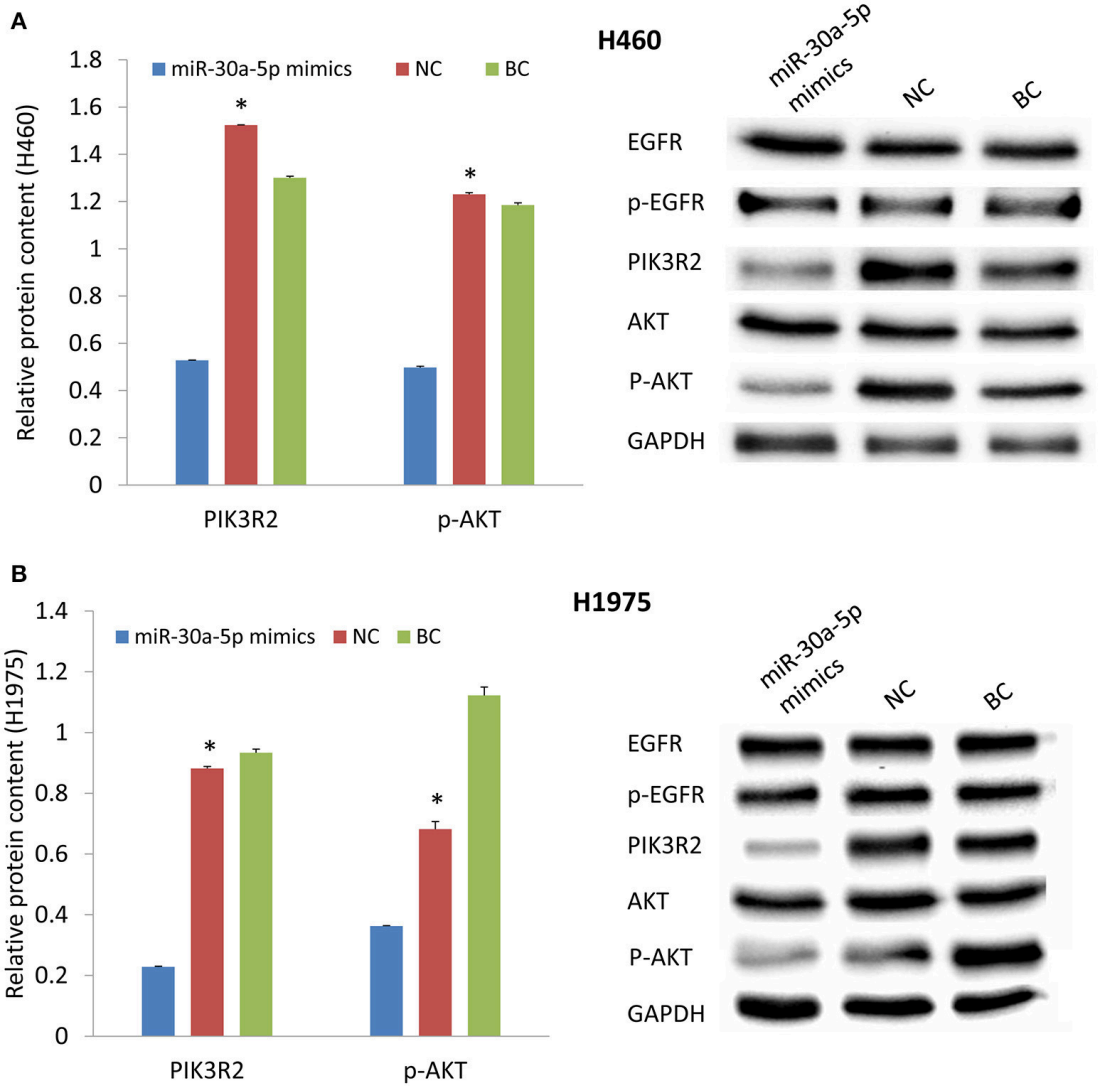
**Effect of miR-30a-5p mimics on the protein expression levels of EGFR, PIK3R2, and AKT**. **(A)** Protein expression in H460 cell line. **(B)** Protein expression in H1975 cell line. GAPDH was used as an internal control. The chart results were presented as the mean of three independent experiments. Blots were the representative of three independent experiments. ^*^P < 0.05, one-way ANOVA used to compare three groups.

**Figure 4:**
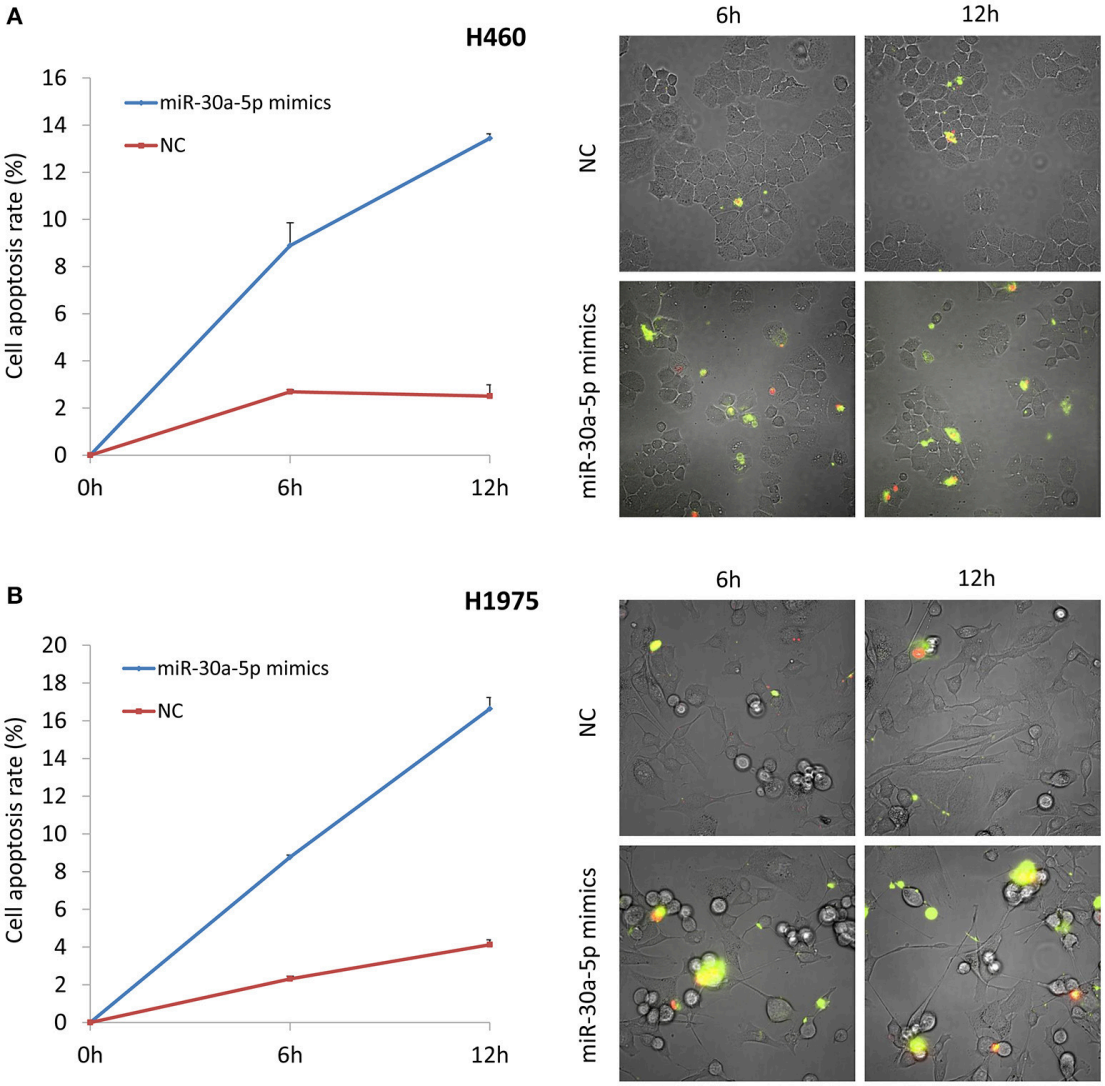
**Cell apoptosis assay after miR-30a-5p mimics transfected at 6 and 12 h**. **(A)** Cell apoptosis assay in H460 cell line. **(B)** Cell apoptosis assay in H1975 cell line. The chart results were presented as the mean of three independent experiments. Representative microscope images were given. Green color indicates the early stage of cell apoptosis, and the red color represents the apoptotic cells at the late stage.

MiR-30a-5p mimics were further transfected to H460 and H1975 cells. Treatment with miR-30a-5p mimics significantly decreased the expression level of PIK3R2 and p-AKT compared to the negative and blank control groups from Tukey's *post-hoc* test (*p* < 0.05; Figure 3). The cell apoptosis assay was further performed to demonstrate the effect of miR-30a-5p mimics on the apoptosis in both H460 and H1975 cells. The microscope was used to detect the apoptosis signals and take images at 6 and 12 h after adding Annexin V-FITC. The cell apoptosis rate was then calculated accordingly. Our results revealed that the cell apoptosis rate was significantly increased at both 6 and 12 h after miR-30a-5p mimics transfection when compared to the negative control group in both H460 and H1975 cells (*p* < 0.05; Figure 4).

### Treatment with miR-30a-5p mimics inhibits cell invasion and migration

**Figure 5:**
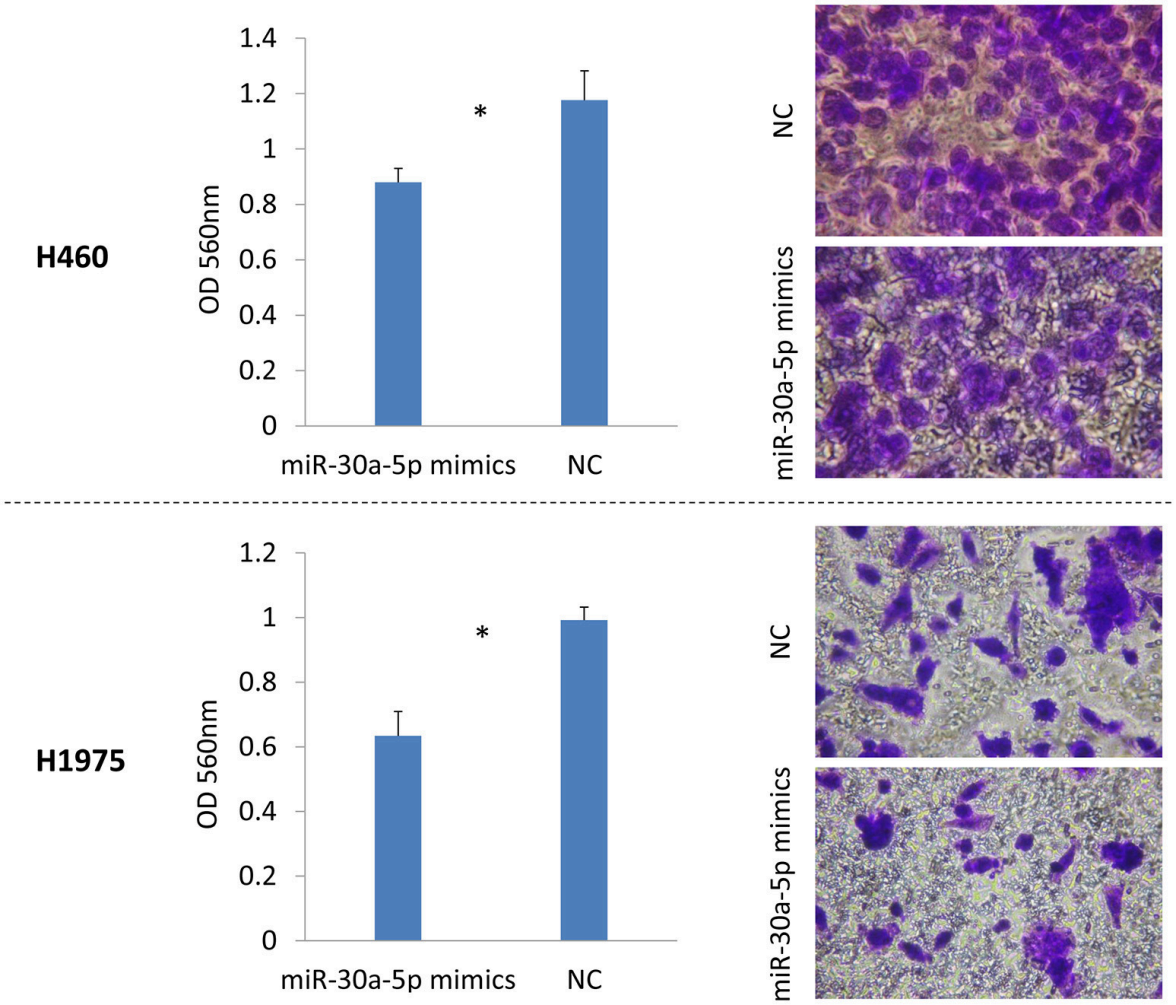
**Cell invasion assay after miR-30a-5p mimics transfected in H460 and H1975 cell lines**. Invasive cells on the bottom of the invasion membrane were stained and quantified at OD 560 nm after extraction. The chart results were presented as the mean of three independent experiments. ^*^P < 0.05, *t*-test used to compare two groups.

**Figure 6:**
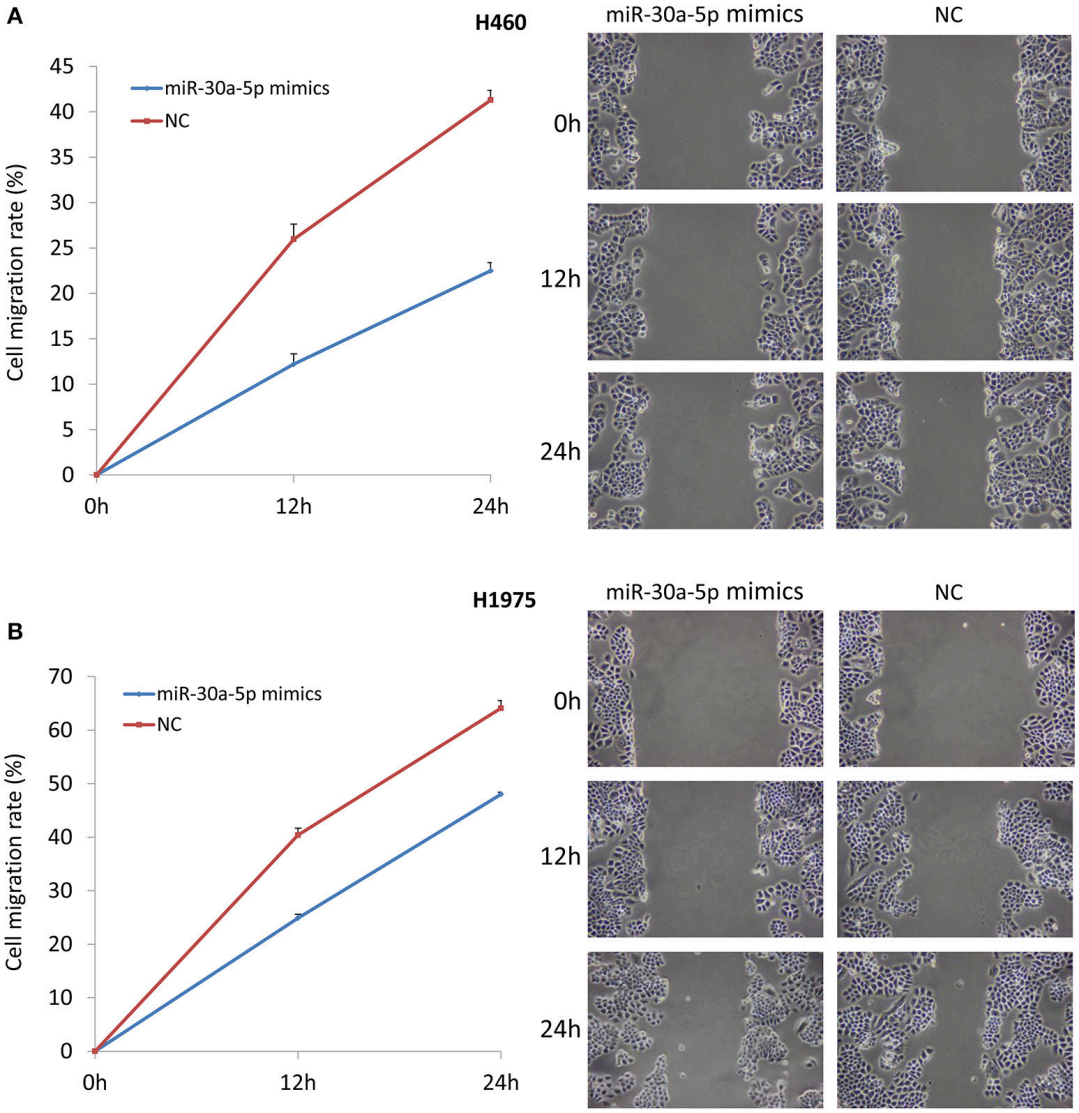
**Cell wound healing assay after miR-30a-5p mimics transfection**. Cell migrating to the scratch was monitored and took images at 0, 12, and 24 h after given the wound using microscope. **(A)** Cell wound healing assay in H460 cell line. **(B)** Cell wound healing assay in H1975 cell line. The chart results were presented as the mean of three independent experiments. Representative microscope images of wound healing were given. The wound length was used to calculate the migration rate.

We also tested the invasion and migration properties after the transfection of miR-30a-5p mimics. The microscope was used to take images for cell invasion. The invasion ability was evaluated based on the OD 560 nm value. The results demonstrated that the invasive cells were significantly decreased in the miR-30a-5p mimics transfected group compared to the negative control group in both H460 and H1975 cells (Figure 5). Cell migration was detected and imaged using microscope at 0, 12, and 24 h after the wound to investigate the role of miR-30a-5p in cell migration. Notably, the cell migration rate was significantly decreased at both 12 and 24 h when treated with miR-30a-5p mimics compared to the negative control group in both H460 and H1975 cells (*p* < 0.05; Figure 6).

## Discussion

In this study, we explored the miR-30a-5p roles in overcoming the drug resistance in the EGFR and IGF-1R signaling pathways in two Gefitinib-resistant NSCLC cell lines, NCI-H460 and NCI-H1975. IGF-1R is one of the tyrosine kinase receptors that share the same downstream molecules with EGFR. Researchers have found that IGF-1R can activate the shared PI3K/AKT signaling pathway (Zhou et al., 2015). Dual inhibition of EGFR and IGF-1R was done to reverse the drug resistance and test PI3K/AKT signaling pathway. We further inhibited the expression of PI3K through shRNA to investigate its phosphorylated downstream molecule p-AKT expression level. MiR-30a-5p mimics were transfected into cells to verify its role in cancer cell apoptosis, invasion and migration properties and the signaling pathway.

Dual inhibition of EGFR and IGF-1R could block the PI3K/AKT signaling pathway. We found that EGFR inhibitor by its own could not reduce the expression level of p-AKT, but the combination of EGFR inhibitor and IGF-1R inhibitor significantly lowered the p-AKT levels compared to the control group (Figure 1). The PI3K/AKT pathway is one of the major signaling networks that transduce the activated EGFR signals, leading to cell proliferation, and survival (Sharma et al., 2007; Gandhi et al., 2009). We found that treatment with PIK3R2-shRNA significantly decreased the expression level of p-AKT (Figure 2). Knockdown of PI3K had the same effect with the dual inhibition of EGFR and IGF-1R to reduce the expression of the phosphorylated downstream molecule p-AKT in the signaling pathway, indicating that PI3K could be regarded as a potential drug target for TKI-resistant NSCLC treatment. PI3K is a kind of enzyme that phosphorylates phosphatidylinositol biphosphate to phosphatidylinositol triphosphate, which is highly activated by receptor tyrosine kinase signaling molecules, including EGFR and IGF-1R (Kurosu et al., 1997; Roche et al., 1998; Vanhaesebroeck and Waterfield, 1999; Cantley, 2002; Zito et al., 2012). Phosphatidylinositol triphosphate can activate AKT to mediate protein synthesis, cell growth and survival (Engelman et al., 2006; Zito et al., 2012). It has been found that PI3K might be a valuable drug target (Zito et al., 2012). Dual inhibition of PI3K and mTOR was synergistic in the NSCLC cell lines *in vitro* and further leaded to cell growth inhibition and apoptosis (Zito et al., 2012). Combining PI3K and mTOR inhibitors has been verified the promising activity in various malignancies (Serra et al., 2008; Brachmann et al., 2009; Aziz et al., 2010).

The dysregulation of miRNAs makes a great contribution to cancer development and cancer drug resistance (Morgillo et al., 2006; Yeo et al., 2015). It has been reported that miR-30b, miR-30c, miR-221, and miR-222 affect the Gefitinib-induced apoptosis and epithelial-mesenchymal transition of NSCLC cells by inhibiting the expression of some important oncogenes (Yeo et al., 2015). MiR-30a-5p is predicted to regulate PIK3R2 by TargetScan and PicTar, which was also found to be negatively associated with the expression of PIK3R2 from microarray data analysis (Wang et al., 2016). We identified that treatment with miR-30a-5p mimics could significantly inhibit PIK3R2 expression to further reduce the level of p-AKT in cell lines (Figure 3). We also investigated the role of miR-30a-5p in cell properties. Overexpression of miR-30a-5p could induce cell apoptosis, as well as inhibit cell invasion and migration (Figures 4–6). MiRNAs are remarkable for drug discovery in cancer treatment. Researchers have tested a liposomal nanoparticle loaded with synthetic miRNA-34a mimics (MRX34) against cancer cells combined with the EGFR-TKI Erlotinib (Bader, 2012; Zhao et al., 2014).

In this study, we identified that dual inhibition of EGFR and IGF-1R could block the PI3K/AKT signaling pathway. NSCLC cell line H1975 has the T790M EGFR mutation and is resistant to EGFR-TKIs, such as Gefitinib and Erlotinib (Pao et al., 2005; Tandon et al., 2011). The activation of IGR-1R signaling pathway may be a novel mechanism that leading to drug resistance in H1975, except for EGFR mutation. The same findings were also identified in another Gefitinib-resistant NSCLC cell line, H460. Knockdown of the shared downstream molecule PI3K could decrease the expression level of p-AKT, indicating PI3K may be a potential drug target for cancer treatment. Overexpression of miR-30a-5p could significantly reduce the PI3K regulatory subunit PIK3R2 expression to further induce cell apoptosis, inhibit cell invasion and migration properties. Hence, combination of miR-30a-5p and other EGFR-TKIs may increase the cancer cell sensitivity to targeted drugs and provide a novel approach for NSCLC treatment.

## Author contributions

LC and FM initiated the project and participated in its design. FM and FW performed laboratory experiments and data analysis. FW, LW, SW, and WC participated in the experimental design and coordination of the study. FM was responsible for writing the manuscript. All the authors were involved in discussion and editing of the manuscript.

### Conflict of interest statement

The authors declare that the research was conducted in the absence of any commercial or financial relationships that could be construed as a potential conflict of interest.

## References

[B1] Agarwal V. Bell G. W. Nam J. W. Bartel D. P. (2015). Predicting effective microRNA target sites in mammalian mRNAs. Elife 4:e05005. 10.7554/eLife.05005 26267216 PMC4532895

[B2] Aziz S. A. Jilaveanu L. B. Zito C. Camp R. L. Rimm D. L. Conrad P.. (2010). Vertical targeting of the phosphatidylinositol-3 kinase pathway as a strategy for treating melanoma. Clin. Cancer Res. 16, 6029–6039. 10.1158/1078-0432.CCR-10-1490 21169255 PMC3058635

[B3] Bader A. G. (2012). miR-34 - a microRNA replacement therapy is headed to the clinic. Front. Genet. 3:120. 10.3389/fgene.2012.00120 22783274 PMC3387671

[B4] Brachmann S. M. Hofmann I. Schnell C. Fritsch C. Wee S. Lane H.. (2009). Specific apoptosis induction by the dual PI3K/mTor inhibitor NVP-BEZ235 in HER2 amplified and PIK3CA mutant breast cancer cells. Proc. Natl. Acad. Sci. U.S.A. 106, 22299–22304. 10.1073/pnas.0905152106 20007781 PMC2799764

[B5] Breuer R. H. Postmus P. E. Smit E. F. (2005). Molecular pathology of non-small-cell lung cancer. Respiration 72, 313–330. 10.1159/000085376 15942304

[B6] Camp E. R. Summy J. Bauer T. W. Liu W. Gallick G. E. Ellis L. M. (2005). Molecular mechanisms of resistance to therapies targeting the epidermal growth factor receptor. Clin. Cancer Res. 11, 397–405. 15671571

[B7] Cantley L. C. (2002). The phosphoinositide 3-kinase pathway. Science 296, 1655–1657. 10.1126/science.296.5573.1655 12040186

[B8] Chan S. Han K. Qu R. Tong L. Li Y. Zhang Z.. (2015). 2,4-Diarylamino-pyrimidines as kinase inhibitors co-targeting IGF1R and EGFRL^858R/T790M^. Bioorg. Med. Chem. Lett. 25, 4277–4281. 10.1016/j.bmcl.2015.07.089 26259806

[B9] Davalos V. Moutinho C. Villanueva A. Boque R. Silva P. Carneiro F.. (2012). Dynamic epigenetic regulation of the microRNA-200 family mediates epithelial and mesenchymal transitions in human tumorigenesis. Oncogene 31, 2062–2074. 10.1038/onc.2011.383 21874049 PMC3330264

[B10] Engelman J. A. Luo J. Cantley L. C. (2006). The evolution of phosphatidylinositol 3-kinases as regulators of growth and metabolism. Nat. Rev. Genet. 7, 606–619. 10.1038/nrg1879 16847462

[B11] Gandhi J. Zhang J. Xie Y. Soh J. Shigematsu H. Zhang W.. (2009). Alterations in genes of the EGFR signaling pathway and their relationship to EGFR tyrosine kinase inhibitor sensitivity in lung cancer cell lines. PLoS ONE 4:e4576. 10.1371/journal.pone.0004576 19238210 PMC2642732

[B12] Guevara-Aguirre J. Balasubramanian P. Guevara-Aguirre M. Wei M. Madia F. Cheng C. W.. (2011). Growth hormone receptor deficiency is associated with a major reduction in pro-aging signaling, cancer, and diabetes in humans. Sci. Transl. Med. 3, 70ra13. 10.1126/scitranslmed.3001845 21325617 PMC3357623

[B13] Ho J. C. (2011). Targeted therapy for non-small cell lung cancer. Med. Bull. 16, 19–21. 23425904

[B14] Krek A. Grün D. Poy M. N. Wolf R. Rosenberg L. Epstein E. J.. (2005). Combinatorial microRNA target predictions. Nat. Genet. 37, 495–500. 10.1038/ng1536 15806104

[B15] Kurosu H. Maehama T. Okada T. Yamamoto T. Hoshino S. Fukui Y.. (1997). Heterodimeric phosphoinositide 3-kinase consisting of p85 and p110β is synergistically activated by the βγ subunits of G proteins and phosphotyrosyl peptide. J. Biol. Chem. 272, 24252–24256. 10.1074/jbc.272.39.24252 9305878

[B16] Morgillo F. Woo J. K. Kim E. S. Hong W. K. Lee H. Y. (2006). Heterodimerization of insulin-like growth factor receptor/epidermal growth factor receptor and induction of survivin expression counteract the antitumor action of erlotinib. Cancer Res. 66, 10100–10111. 10.1158/0008-5472.CAN-06-1684 17047074

[B17] Pao W. Miller V. A. Politi K. A. Riely G. J. Somwar R. Zakowski M. F.. (2005). Acquired resistance of lung adenocarcinomas to gefitinib or erlotinib is associated with a second mutation in the EGFR kinase domain. PLoS Med. 2:e73. 10.1371/journal.pmed.0020073 15737014 PMC549606

[B18] Peled N. Wynes M. W. Ikeda N. Ohira T. Yoshida K. Qian J.. (2013). Insulin-like growth factor-1 receptor (IGF-1R) as a biomarker for resistance to the tyrosine kinase inhibitor gefitinib in non-small cell lung cancer. Cell Oncol (Dordr) 36, 277–288. 10.1007/s13402-013-0133-9 23619944 PMC4186686

[B19] Roche S. Downward J. Raynal P. Courtneidge S. A. (1998). A function for phosphatidylinositol 3-kinase β (p85α-p110β) in fibroblasts during mitogenesis: requirement for insulin- and lysophosphatidic acid-mediated signal transduction. Mol. Cell. Biol. 18, 7119–7129. 10.1128/MCB.18.12.7119 9819398 PMC109293

[B20] Serra V. Markman B. Scaltriti M. Eichhorn P. J. Valero V. Guzman M.. (2008). NVP-BEZ235, a dual PI3K/mTOR inhibitor, prevents PI3K signaling and inhibits the growth of cancer cells with activating PI3K mutations. Cancer Res. 68, 8022–8030. 10.1158/0008-5472.CAN-08-1385 18829560

[B21] Sharma S. V. Bell D. W. Settleman J. Haber D. A. (2007). Epidermal growth factor receptor mutations in lung cancer. Nat. Rev. Cancer 7, 169–181. 10.1038/nrc2088 17318210

[B22] Tandon R. Kapoor S. Vali S. Senthil V. Nithya D. Venkataramanan R.. (2011). Dual epidermal growth factor receptor (EGFR)/insulin-like growth factor-1 receptor (IGF-1R) inhibitor: a novel approach for overcoming resistance in anticancer treatment. Eur. J. Pharmacol. 667, 56–65. 10.1016/j.ejphar.2011.04.066 21640718

[B23] Vanhaesebroeck B. Waterfield M. D. (1999). Signaling by distinct classes of phosphoinositide 3-kinases. Exp. Cell Res. 253, 239–254. 10.1006/excr.1999.4701 10579926

[B24] Wang F. Meng F. Wang L. Wong S. C. C. Cho W. C. S. Chan L. W. C. (2016). Associations of mRNA:microRNA for the Shared Downstream Molecules of EGFR and Alternative Tyrosine Kinase Receptors in Non-small Cell Lung Cancer. Front. Genet. 7:173. 10.3389/fgene.2016.00173 27790245 PMC5061729

[B25] Yeo C. D. Park K. H. Park C. K. Lee S. H. Kim S. J. Yoon H. K.. (2015). Expression of insulin-like growth factor 1 receptor (IGF-1R) predicts poor responses to epidermal growth factor receptor (EGFR) tyrosine kinase inhibitors in non-small cell lung cancer patients harboring activating EGFR mutations. Lung Cancer 87, 311–317. 10.1016/j.lungcan.2015.01.004 25617986

[B26] Yu H. A. Sima C. S. Huang J. Solomon S. B. Rimner A. Paik P.. (2013). Local therapy with continued EGFR tyrosine kinase inhibitor therapy as a treatment strategy in EGFR-mutant advanced lung cancers that have developed acquired resistance to EGFR tyrosine kinase inhibitors. J. Thorac. Oncol. 8, 346–351. 10.1097/JTO.0b013e31827e1f83 23407558 PMC3673295

[B27] Zhang Z. Lee J. C. Lin L. Olivas V. Au V. LaFramboise T.. (2012). Activation of the AXL kinase causes resistance to EGFR-targeted therapy in lung cancer. Nat. Genet. 44, 852–860. 10.1038/ng.2330 22751098 PMC3408577

[B28] Zhao J. Kelnar K. Bader A. G. (2014). In-depth analysis shows synergy between erlotinib and miR-34a. PLoS ONE 9:e89105. 10.1371/journal.pone.0089105 24551227 PMC3925231

[B29] Zhou J. Wang J. Zeng Y. Zhang X. Hu Q. Zheng J.. (2015). Implication of epithelial-mesenchymal transition in IGF1R-induced resistance to EGFR-TKIs in advanced non-small cell lung cancer. Oncotarget 6, 44332–44345. 10.18632/oncotarget.6293 26554308 PMC4792560

[B30] Zito C. R. Jilaveanu L. B. Anagnostou V. Rimm D. Bepler G. Maira S. M.. (2012). Multi-level targeting of the phosphatidylinositol-3-kinase pathway in non-small cell lung cancer cells. PLoS ONE 7:e31331. 10.1371/journal.pone.0031331 22355357 PMC3280285

